# Two-Photon Absorption Cross-Sections in Fluorescent Proteins Containing Non-canonical Chromophores Using Polarizable QM/MM

**DOI:** 10.3389/fmolb.2020.00111

**Published:** 2020-06-12

**Authors:** Maria Rossano-Tapia, Jógvan Magnus Haugaard Olsen, Alex Brown

**Affiliations:** ^1^Department of Chemistry, University of Alberta, Edmonton, AB, Canada; ^2^Hylleraas Centre for Quantum Molecular Sciences, Department of Chemistry, UiT The Arctic University of Norway, Tromsø, Norway

**Keywords:** QM/MM, ONIOM, polarizable embedding, two-photon absorption, chromophores, fluorescent proteins, protein design, non-canonical amino acids

## Abstract

Multi-photon absorption properties, particularly two-photon absorption (2PA), of fluorescent proteins (FPs) have made them attractive tools in deep-tissue clinical imaging. Although the diversity of photophysical properties for FPs is wide, there are some caveats predominant among the existing FP variants that need to be overcome, such as low quantum yields and small 2PA cross-sections. From a computational perspective, Salem et al. (2016) suggested the inclusion of non-canonical amino acids in the chromophore of the red fluorescent protein DsRed, through the replacement of the tyrosine amino acid. The 2PA properties of these new non-canonical chromophores (nCCs) were determined in vacuum, i.e., without taking into account the protein environment. However, in the computation of response properties, such as 2PA cross-sections, the environment plays an important role. To account for environment and protein–chromophore coupling effects, quantum mechanical/molecular mechanical (QM/MM) schemes can be useful. In this work, the polarizable embedding (PE) model is employed along with time-dependent density functional theory to describe the 2PA properties of a selected set of chromophores made from non-canonical amino acids as they are embedded in the DsRed protein matrix. The objective is to provide insights to determine whether or not the nCCs could be developed and, thus, generate a new class of FPs. Results from this investigation show that within the DsRed environment, the nCC 2PA cross-sections are diminished relative to their values in vacuum. However, further studies toward understanding the 2PA limit of these nCCs using different protein environments are needed.

**Graphical Abstract:**
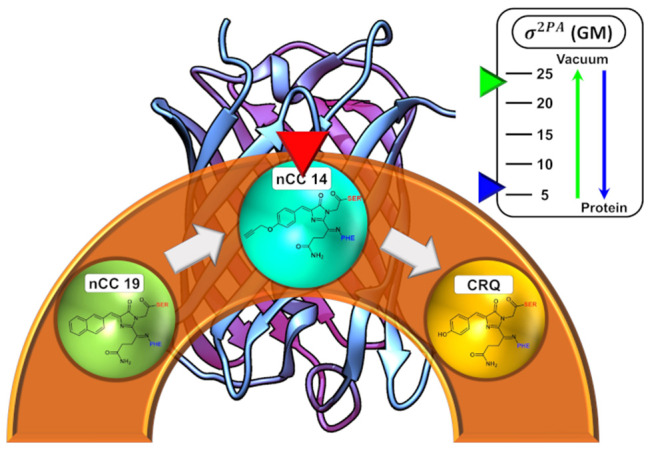
Depiction of the the dependence of the 2PA cross-sections for an nCC in vacuum versus the protein environment.

## 1. Introduction

**Figure 1:**
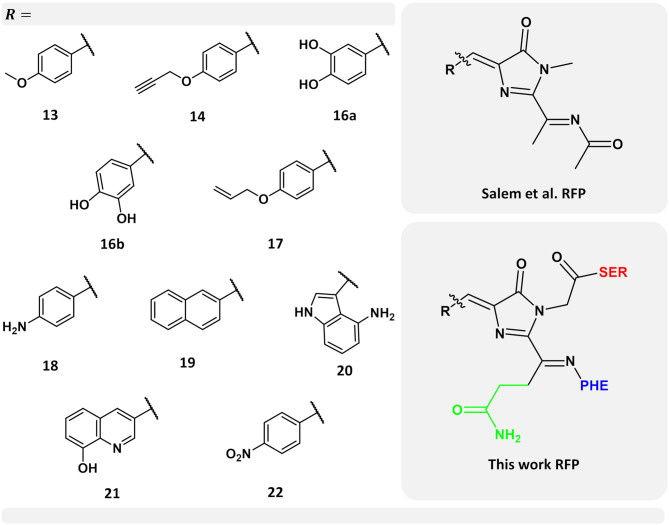
Left-hand side: selected set of chromophores from the work carried out by Salem et al. (2016). Numerical labels are the same as in their work for ease of comparison. Top right-hand side corner: chromophore model used by Salem et al. (2016) Bottom right-hand side corner: chromophore model used in this work. In green, the chromophore side chain -CH_2_-CH_2_-CO-NH_2_ excluded in the previous work (Salem et al., 2016), while neighboring amino acids serine and phenylalanine are indicated in red and blue, respectively.

The work of Shimomura and co-workers on the bioluminescent crystal jellyfish (Shimomura, 2005) had among its principal outcomes the discovery of the green fluorescent protein (GFP) (Shimomura et al., 1962), a barrel-shaped protein in which a chromophore (Shimomura, 1979; Cody et al., 1993) is located and responsible for its bright green color. The presence of such a barrel-shaped protein was later discovered not to be exclusive to the crystal jellyfish; indeed, similar fluorescent proteins (FPs) were also found among corals and some other species of the Anthozoa class. FPs of the Anthozoa species exhibit a red-shift in their absorption and emission properties with respect to their GFP homologs, and therefore they were called red fluorescent proteins (RFPs). One example is the DsRed RFP, which is found in the anemone Discosoma striata (Matz et al., 1999). The chromophore structure in FPs is characterized by an imidazole ring, made from the cyclization of three amino acids, which in the case of DsRed are glutamine 66, tyrosine 67, and glycine 68 (Gln66, Tyr67, and Gly68, respectively). The RFP chromophore structure is shown in Figure 1 (bottom right-hand side corner).

Fluorescent proteins have been used as dyes and clinical markers over the last two decades and their multi-photon absorption properties have allowed them to be applied in deep-tissue clinical imaging at low phototoxicity (Chudakov et al., 2010; Drobizhev et al., 2011). Currently, dozens of hues of FPs covering all the colors in the visible spectrum have been engineered, with the purpose of overcoming some of the most common limitations of many FPs, such as low quantum yield, autobleaching, phototoxicity, and weak two-photon absorption (measured in terms of the cross-section, σ^2PA^). Some strategies in the design of new FPs involve changing the amino acids in the protein sequence, like the way mRFP1 (a monomeric DsRed variant) was tailored trying to improve DsRed properties, and from which the so-called fruit series was obtained by Shaner et al. (2004). However, in 2015 and 2016, modification of GFP (Salem and Brown, 2015) and RFP (Salem et al., 2016) chromophores, through the substitution of the tyrosine in these chromophores by one of a selected set of non-canonical amino acids previously obtained by Liu and Schultz (2010), was suggested as a means toward improving two-photon absorption. The resulting chromophores from the incorporation of non-canonical amino acids are here called non-canonical chromophores (nCCs). Incorporation of nCCs has been accomplished and studied previously by experimental means as discussed for example by Fang et al. (2018) and Budisa and Pal (2004). Regarding FPs, gold fluorescent protein (Bae et al., 2003) is an example of inclusion of non-canonical amino acids for which one-photon absorption properties have been measured experimentally and explored computationally, as will be discussed later in this text.

Beyond expanding the color span or improving the features of existing FPs, the persistent efforts on creating novel FPs are motivated by their utility as bioimaging tools (Zhao and Campbell, 2015), including the fact that they do not require any accessorial proteins or fluorophores as their fluorescence comes from the chromophore embedded in them.

One- and two-photon absorption (1PA and 2PA, respectively) properties of FPs and their chromophores have been addressed using different computational tools, mainly quantum mechanical (QM) methods based on time-dependent density functional theory (TD-DFT) (Nifosì et al., 2007; Nifosì and Luo, 2007a,b; Salem and Brown, 2014; Salem et al., 2017). Due to its computational cost, TD-DFT can only be applied to small systems, i.e., the chromophore and, possibly, including a few nearby residues, rather than to the entire chromophore-protein system. Outcomes of computational investigations have been compared to experimental data and shown to provide reasonable agreement (Voityuk et al., 1998; Nifosì et al., 2007; Nifosì and Luo, 2007b), and therefore many 1PA and 2PA studies have been carried out for the FP chromophores in vacuum without considering the role of the protein matrix, for example the work by Nifosì et al. (2007); Nifosì and Luo (2007a), Nifosì and Luo (2007b), Filippi et al. (2009); List et al. (2012a); Salem and Brown (2014), Salem and Brown (2015), Beerepoot et al. (2015), Salem et al. (2016), among others. Other studies have included and/or examined the role of the protein in the computation of 1PA and 2PA properties of FPs using combined quantum mechanical/molecular mechanical methods (QM/MM), that includes work by Marques et al. (2003), Sanchez-Garcia et al. (2009), Sanchez-Garcia et al. (2010), Steindal et al. (2012), Steindal et al. (2016), List et al. (2012b), Kaila et al. (2013), Beerepoot et al. (2014a), Schwabe et al. (2015), Nåbo et al. (2017), Kulakova et al. (2018), Moron et al. (2019), some of which have shown that the protein environment can have a large impact on one- and multi-photon absorption properties (List et al., 2012b; Beerepoot et al., 2014a; Schwabe et al., 2015; Steindal et al., 2016).

QM/MM approaches involve sectioning the entire system into different parts (two or more layers) where each part is modeled using a different computational approach. The part of the system where bond-breaking or -forming takes place, or excited-state processes occur, is described through QM methods. The rest of the layer(s) can be treated using an MM model or a less rigorous (computationally less expensive) QM method. QM/MM methods are used to reduce the cost of computations of large systems that would not be feasible to study using pure QM means with current computational resources.

In particular, for the set of nCCs mentioned above, previous attempts of addressing the environment effects on their 2PA properties have been made using the self-consistent reaction field (SCRF) polarizable continuum model (PCM) (Salem et al., 2016). Other approaches have used molecular dynamics (MD) along with QM computations on the chromophore alone, such as the study carried out by Şimşek and Brown (2018) which examined 1PA and 2PA properties of gold fluorescent protein (Bae et al., 2003). However, continuum models, such as PCM, are not suitable for heterogeneous systems like proteins, and while QM computations on the chromophore carried out on top of MD simulations capture some indirect environment effects through the geometry, they lack the direct electrostatic interactions. The latter can be efficiently and accurately included using the polarizable embedding (PE) model (Olsen et al., 2010; Olsen and Kongsted, 2011), which has also been used to compute 2PA properties of FPs (List et al., 2012b, 2016; Steindal et al., 2016). The PE model is a fragment-based quantum–classical approach similar to QM/MM, but where the MM part is subdivided into small fragments (usually amino acid residues in the case of proteins), that are represented by atom-centered charges, dipoles, and higher-order multipoles, as well as polarizabilities. These parameters are derived from QM calculations on the individual fragments which thus leads to a very high-quality representation of the protein (Olsen et al., 2015). The PE model was designed to describe general response properties, including 2PA. It allows the QM and MM parts of the system to mutually polarize each other, and can also account for local-field effects.

The local field acting on a chromophore that is embedded in a polarizable environment is generally different from the externally applied field due to the polarization of the environment by the external field. In the PE model, this effect is modeled through the so-called external effective field (EEF) approach (List et al., 2016). The EEF effects will not affect the excitation energies but can be important for other properties, such as multi-photon absorption. In fact, studies carried out on the DsRed and GFP proteins, showed that local-field effects play a major role in the determination of multi-photon cross-sections (List et al., 2016; Steindal et al., 2016; Reinholdt et al., 2017). Indeed, the results showed that inclusion of local-field effects, through EEF, is required in order to be comparable to full QM results (List et al., 2016). The failure to include local-field effects in the computation of 2PA cross-sections can lead to a misrepresentation of 2PA features of FPs, as shown by Steindal et al. (2016) in the case of GFP. This topic is discussed further later in this work.

Using the PE model, including local-field effects, we investigated the 1PA and 2PA properties of a selected set of ten nCCs taken from a more complete set previously studied by Salem et al. (2016). The latter with the objective of providing further insights into the properties of these nCCs within a more realistic context and to provide more information of the performance of the PE model in the computation of multi-photon absorption properties in FPs. Figure 1 shows the models used here, where -R represents each of the non-canonical amino acids employed. Figure 1 also shows the differences between the previous chromophore model (Salem et al., 2016) and the model used in this work; further discussion about this matter will be provided in the next sections. The selected set of chromophores used here (Figure 1) are those which in Salem et al. (2016) exhibited the largest 2PA cross-sections and the largest intrinsic cross-section (obtained from the analysis of σ^2PA^ with respect to the tilt and twist angles) as in the case of nCC 21.

## 2. Computational Methods

### 2.1. Modeling the Protein-Chromophore Structures

The main challenge in the construction of nCC-protein model structures is the fact that none of the nCCs shown in Figure 1 have been matured in a red fluorescent-type protein experimentally. Thus, there are no experimental protein crystal structures that can be used either directly or as initial structures for geometry optimization. Only one of the nCCs (no. 20) has been successfully expressed in the gold fluorescent protein and its 1PA properties have been evaluated (Bae et al., 2003). However, this protein belongs to the family of GFP derivatives. To overcome this shortcoming, the construction of nCC-protein models consisted of two stages: (i) selecting a protein structure that can be used as a host for the nCC based on the criteria that the host protein should exhibit red-shifted absorption (like RFPs) and possess a considerable (for FPs) 2PA cross-section (>50 GM), and (ii) replacing the canonical amino acid chromophore with each one of the nCCs shown in Figure 1.

**Figure 2:**
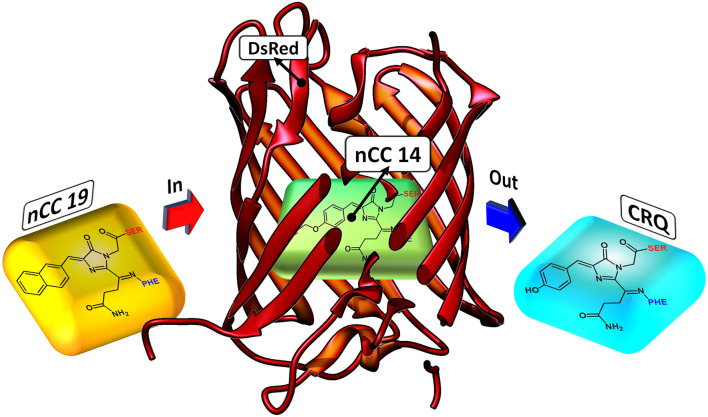
Depiction of how nCC-DsRed (1ZGO) models were created. The native chromophore (CRQ) in the DsRed protein matrix is replaced by the non-canonical chromophore (nCC) model.

TagRFP (Merzlyak et al., 2007) and some members of the fruit series (Shaner et al., 2004) are among the brightest of the RFP family (Chudakov et al., 2010). However, we used DsRed (PDB: 1ZGO) (Tubbs et al., 2005) because this protein meets the criteria described above. It is the parent protein of the most common RFPs and has not been tailored in the laboratory around any particular chromophore, in the way the fruit series proteins were tailored around the native DsRed chromophore (CRQ), see Figure 2. Moreover, the 2PA properties of DsRed have previously been studied computationally through QM/MM schemes (Sanchez-Garcia et al., 2009, 2010; List et al., 2012b, 2016) and this provides us with a reference to which we can compare the data we obtain here. Experimentally, Drobizhev et al. (2011) reported the DsRed 2PA cross-section to be 103 GM for the long-wave absorption band (1, 050 nm). To build the nCC-DsRed models, we used a single monomer of 1ZGO, and modified the native CRQ chromophore to each of the nCCs. A depiction of how the models are structured is shown in Figure 2. Missing hydrogens in 1ZGO were added using pdb4amber (Case et al., 2018), whereas the missing residues 1–5, which are located outside the barrel of the protein, were not added since they were not considered crucial for the purpose of this work. The present work represents an initial computational investigation of the protein effect on 2PA cross-sections in RFP-like nCCs. It is thus based on a single geometry-optimized structure for each chromophore, i.e., no conformational sampling is included. This way we get an estimate of the effect of adding the protein matrix into the computation of 2PA cross-sections of the non-canonical amino acids studied here. Moreover, we did not include water molecules inside the cavity or solvent molecules around the protein, because understanding the presence of water would require molecular dynamics simulations. The inclusion of statistical sampling could change the quantitative results of the present work. However, this is beyond the scope of the present work but could be the subject of future research.

The nCC-DsRed structures were optimized using a two-layer ONIOM (Dapprich et al., 1999; Vreven et al., 2006; Clemente et al., 2010) QM/MM scheme implemented in Gaussian 16 (Frisch et al., 2016). The QM layer comprised the nCC and the phenylalanine residue bonded to it. The whole phenylalanine amino acid structure was included to avoid cutting double bonds or bonds near the conjugated chain while retaining the acylimine moiety. Also, the carbonyl group bonded to serine, which was previously excluded by Salem et al. (2016) when studying the isolated chromophores, was included. The rest of the protein is part of the MM layer. All optimizations used mechanical embedding and were carried out in two steps using the default convergence criteria: first, using the semi-empirical PM6 (Stewart, 2007) method; second, using the long-range corrected functional CAM-B3LYP (Yanai et al., 2004) and the 6-31+G(d,p) (Hehre et al., 1972) basis set. Molecular mechanical parameters, including charges, were obtained from the Amber force field libraries (ff96, ff10, and GAFF) included in Amber 18 (Case et al., 2018). In particular, nCC parameters were obtained from the R.E.D. Server (Bayly et al., 1993; Dupradeau et al., 2010; Vanquelef et al., 2011; Wang et al., 2013) using the default type of charges, RESP-A1, and the computational method HF/6-31G(d). Hessian analyses were performed on the optimized geometries in order to verify the absence of imaginary frequencies, and thus that the structure is indeed a minimum.

The CAM-B3LYP functional was chosen based on a comparison of the 14-DsRed model optimized using three different functionals, i.e., CAM-B3LYP, ωB97XD (Chai and Head-Gordon, 2008), and PBE0 (Perdew et al., 1996, 1997), along with the 6-31+G(d,p) basis set, and all within the ONIOM scheme. In all cases, the Amber force field (libraries mentioned above) was used for the MM region. The optimized structures using CAM-B3LYP and ωB97XD did not show substantial structural differences (see Figure S1 comparing the overlapped structures). The PBE0 functional suffered from convergence problems, failing to find a stable minimum. In addition to mechanical embedding, optimization of one of the models, nCC 20, was performed using electrostatic embedding and the CAM-B3LYP functional. In this optimization the residues belonging to the QM region, PHE 60 and CRQ 61, along with residues GLN 59 and SER 62, treated by MM, could relax while the rest of the protein was kept fixed.

### 2.2. Two-Photon Absorption Cross-Section Computations

All computations of 2PA cross-sections were carried out using the Dalton program (Aidas et al., 2014, 2019; Olsen et al., 2018), employing the PE model (Olsen et al., 2010), including effective external field (EEF) effects (List et al., 2016), to describe the protein environment. The 2PA cross-section is given by (Beerepoot et al., 2015)

(1) $$\begin{array}{l} \sigma^{2\text{PA}}=\frac{N\pi^{2}a_{0}^{5}\alpha }{c_{0}}\frac{\omega^{2}}{\Gamma }\delta^{2\text{PA}}\;, \end{array}$$

where *a*_0_ is the Bohr radius, α is the fine structure constant, *c*_0_ is the speed of light, Γ is the lifetime broadening factor, which is derived from a Lorentzian function and assumed to be 3.675·10^−3^ Hartree (or 0.1 eV) to facilitate comparison to experiment (as well as previous computational results), ω is the excitation energy (Hartree/photon), which for 2PA is half the energy difference between the excited and ground states, and δ^2PA^ is the 2PA transition strength. The resulting σ^2PA^ is given in cm^2^ · s · photon^−1^ or GM (Göppert-Mayer after Maria Göppert-Mayer) (Mayer Göppert, 1931).

**Figure 3:**
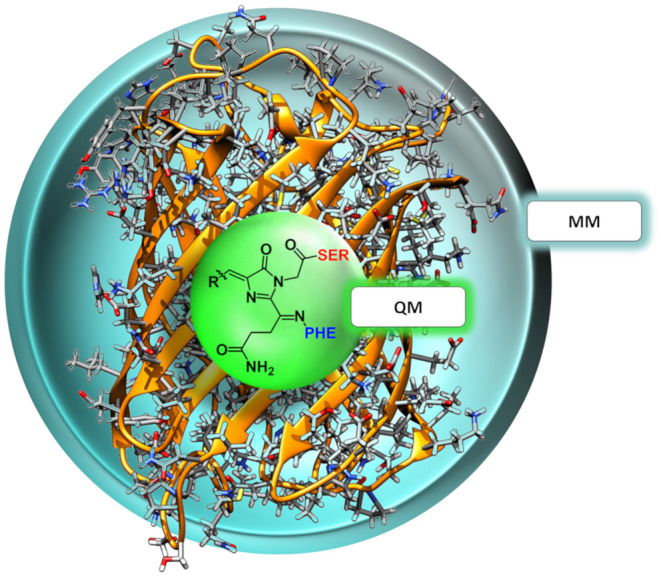
Two-layer QM/MM partitioning in each of the nCC-DsRed systems. In the 2PA computations, the QM region includes the chromophore and the neighboring residues serine (red) and phenylalanine (blue), while the classical MM region includes the protein structure only.

The QM region consisted in all cases mainly of the nCC. Compared to previous models (Salem et al., 2016; Şimşek and Brown, 2018), two changes were made: (i) any side chains in the native CRQ chromophore of DsRed were preserved, and (ii) amino acid residues that are covalently bonded to the chromophore, i.e., serine (SER) and phenylalanine (PHE), were included (see Figure 1). The latter are included in the QM region to avoid cutting any bonds nearby the acylimine moiety, and also to include any possible contributions to the 2PA process that the neighboring amino acids could have (Steindal et al., 2016). For the MM region, corresponding to the rest of the protein, distributed atom-centered charges, dipoles, quadrupoles, and dipole–dipole polarizabilities for each of the amino acid residues were generated using the PyFraME Python package (Olsen, 2018). PyFraME employs Dalton (Aidas et al., 2014, 2019) and LoProp for Dalton (Vahtras, 2014) to compute the parameters based on a fragmentation scheme. The distributed multipoles and polarizabilities were computed using the LoProp approach (Gagliardi et al., 2004) employing the CAM-B3LYP functional and the ANO-form of the 6-31+G* basis set [named loprop-6-31+G(d) in Dalton]. We refer to the work by Steinmann et al. (2019) for a tutorial review on the setup, use, and capabilities of the PE model (Olsen et al., 2010). A commented Python script employing PyFraME used in the present work is included in the repository (Rossano-Tapia et al., 2020). Figure 3 shows a depiction of the QM (SER-nCC-PHE) and MM regions.

**Figure 4:**
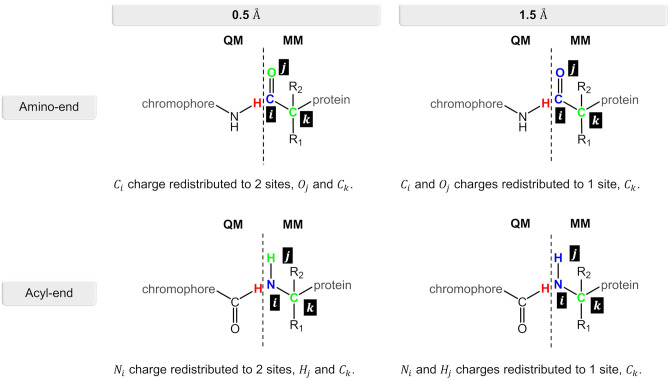
Charge redistribution scheme for the N-terminal and C-terminal sides in the nCC-DsRed systems. Atom(s) in blue represent the charge(s) to be redistributed to the atom site(s) in green.

Computation of 2PA cross-sections was carried out using the CAM-B3LYP functional, while different Pople basis sets [6-31G(d), 6-31+G(d), and 6-31+G(d,p)] and a segmented polarization-consistent basis set (pcseg-2) Jensen (2017), were tested on the 14-DsRed system. After determining the role of the basis set, 2PA cross-sections were computed for the geometry-optimized chromophores (Figure 1) (i) in vacuum (i.e., without the protein), (ii) with charges of the atoms closest to the hydrogen link atoms (0.5 or 1.5 Å) redistributed to nearby atoms to avoid overpolarization due to electronic density–point charge proximity (Figure 4), and (iii) including or excluding EEF effects. Selection of the distances for the charge redistribution was based in Figure S2. For all computations, hydrogen link atoms were treated using the STO-3G minimal basis set to prevent electronic density from “spilling out” and generating spurious orbitals at the bonding sites as described by Steinmann et al. (2019). The outcome from these analyses will be elaborated in the next section.

## 3. Results and Discussion

### 3.1. Geometry Optimization

**Figure 5:**
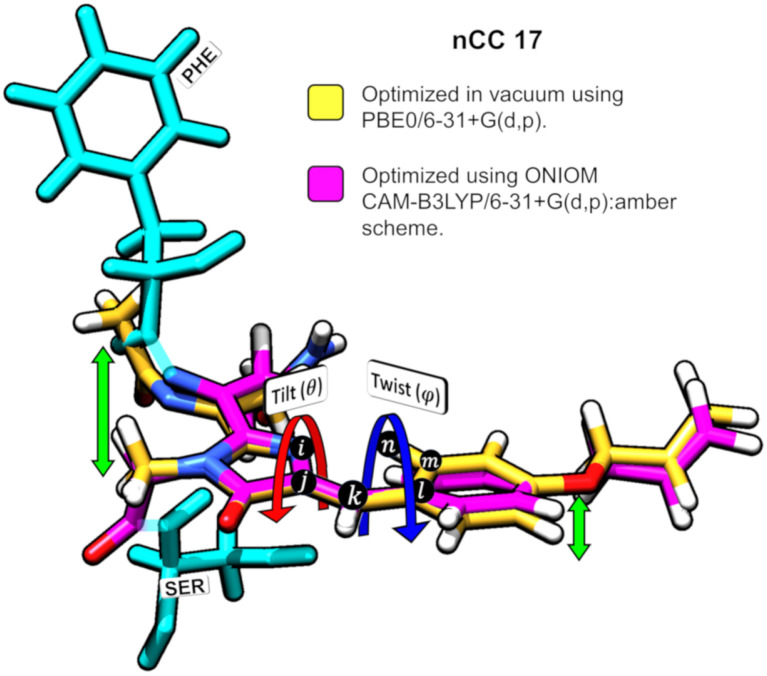
Superposition of nCC 17 structures (a) optimized using PBE0/6-31+G(d,p) Salem et al. (2016) in vacuum and (b) optimized using ONIOM [CAM-B3LYP/6-31+G(d,p):Amber]. Tilt (θ) and twist (ψ) angles are shown. The green arrows indicate the deviation between the structures.

**Figure 6:**
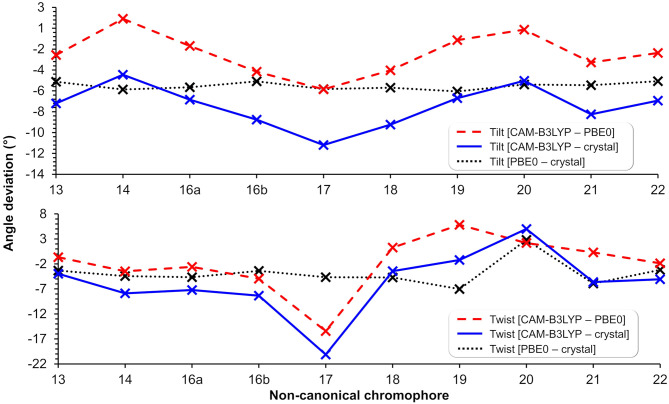
Tilt **(top)** and twist **(bottom)** angle deviations in degrees between structures optimized using CAM-B3LYP/6-31+G(d,p) (in protein), PBE0/6-31+G(d,p) (Salem et al., 2016) (in vacuum), and the canonical chromophore from the crystal structure (PDB ID: 1ZGO) (Tubbs et al., 2005). The lines are included to guide the eye through the deviations of tilt and twist angles within methods and nCC-DsRed models.

The impact of the protein environment on the optimized geometries of the nCCs was determined by comparing each structure obtained using the ONIOM QM/MM CAM-B3LYP/6-31+G(d,p):Amber scheme to the experimental structure of the canonical chromophore in crystal DsRed (Tubbs et al., 2005) and the corresponding non-canonical chromophore optimized in vacuum using PBE0/6-31+G(d,p) Salem et al. (2016). The comparisons are based on the tilt (θ) and twist (φ) dihedral angles, formed by atoms *i*−*j*−*k*−*l* and *k*−*l*−*m*−*n*, respectively, as illustrated in Figure 5. Through computational studies (Salem and Brown, 2015; Salem et al., 2016; Şimşek and Brown, 2018), it has been shown that 2PA cross-sections depend on these angles. Based on experimental data, some authors have observed that θ and φ also play important roles in the chromophore's 1PA properties and in the conjugation of the double bonds found between the chromophore and the acylimine group; RFP family chromophores that exhibit a non-planar structure tend to exhibit smaller quantum yields than, for example, the planar DsRed chromophore (Shu et al., 2006; Pakhomov and Martynov, 2008; Subach and Verkhusha, 2012). The results of the θ and φ angle comparisons are summarized in Figure 6. Table S1 contains the values and deviations of the tilt and twist angles for all nCCs in Figure 1. The relation between the structural differences of the optimized nCCs and their 2PA cross-sections will be discussed in the next subsection.

Apart from applying the ONIOM QM/MM method for the optimization of the nCC-DsRed systems, the QM region used here was also larger than the structures optimized by Salem et al. (2016). In our work, the nCCs and the phenylalanine bonded to it were optimized quantum mechanically; whereas previously only the chromophore structure was optimized, i.e., without any neighboring amino acids. In addition, Salem et al. (2016) excluded the side chain -CH_2_-CH_2_-CO-NH_2_, highlighted in green in Figure 1, and the carbonyl group toward the serine amino acid. These differences in optimization strategy and chromophore definition resulted in differences in the θ and φ angles between the CAM-B3LYP (QM/MM) and PBE0 (vacuum) structures that are between −5.4 and 1.4, and −15.5 and 5.8 degrees, respectively. The deviation of the QM/MM-optimized geometries from the crystal structure range from −10.7 to −4.0 and from −20.1 to −1.2 degrees for θ and φ, respectively. In contrast, the θ and φ angles of the nCCs optimized in vacuum using PBE0 (Salem et al., 2016) do not differ by more than 7 degrees from the crystal structure. Moreover, the vacuum to crystal differences for the θ and φ angles for all nCCs vary within rather small intervals, i.e., between −4.6 and −5.6, and −3.3 and 7.0 degrees, respectively. These small differences in the PBE0-based vacuum structures, along with the larger differences observed for CAM-B3LYP-based QM/MM structures with respect to the crystal structure, suggest that the non-canonical amino acid moiety, -R (Figure 1), does not determine on its own these two angles, and it is less likely that the -R moiety could provide a realistic picture of what the structure of the chromophore in the protein could be without considering the protein in the optimization process. From the differences in the CAM-B3LYP θ angle compared to the PBE0 results and the angles observed experimentally in the crystal, it is clear that θ will be largely impacted by the presence of nearby amino acids and barely by the nature of the -R substituent (Figure 1). The largest CAM-B3LYP to crystal deviations in φ are observed in nCCs 17, 19, 20, and 21, and are likely due to the size of the chromophore. For nCC 17, optimizing it within the volume of the protein cavity played a major role in determining its structure, particularly the θ and φ angles. The embedding of the chromophore in the protein matrix created distortion of the tilt and twist angles in the chromophore as described above. From the protein perspective, the substitution of the canonical chromophore by the non-canonical model caused structural changes in residues non-covalently bonded to it and located in its immediate surroundings. Some of these structural changes included the expansion of the cavity residues away from the chromophore, specifically those residues close to the -R moiety. The structural changes for two selected residues (to ease visualization), SER 139 and LYS 156, in comparison with 1ZGO are shown in Figure S11.

Model nCC 20, one of the nCCs with the largest tilt and twist angle deviations from the DsRed crystal structure and 2PA cross-section computed in vacuum (Salem et al., 2016) and in the protein, was further optimized using electrostatic embedding. The tilt and twist angles of the resulting structure, −1.59 and 11.18 degrees respectively, are similar to the ones in the nCC 20-DsRed structure optimized using mechanical embedding, 0.27 and 8.29 degrees, respectively. Figure S10 illustrates the small changes in the geometry after optimization using electrostatic embedding.

### 3.2. 2PA Cross-Sections

One- and two-photon absorption cross-sections in all nCCs were computed both in vacuum and in protein using PE to model the effects of the protein environment. The QM region in these computations included the chromophore and its two neighboring covalently bonded amino acids, serine and phenylalanine (see Figure 1), whereas the rest of the protein was treated classically. The two charge redistribution schemes depicted in Figure 4 were evaluated using the 14-DsRed model employing CAM-B3LYP and different basis sets [6-31G(d), 6-31+G(d), 6-31+G(d,p), and pcseg-2], in order to establish a suitable approach. The results are provided in Table S2 together with corresponding molecular orbital (MO) plots in Figures S4–S6. For comparison, MO plots of nCC 14 in vacuum are provided in Figure S3.

Using a point-charge redistribution distance of 1.5 Å, results in an unexpected low-intensity transition at around 3.2–3.3 eV, which is most likely due to over-polarization effects. Indeed, an inspection of the MOs (Figure S5) reveals that this is not a relevant transition as the main contribution is from an occupied MO that is not localized on the chromophore. Even the intense transition, which is to the second state, involves a main contribution from an occupied MO that has large components outside of the chromophore. Using instead a redistribution distance of 0.5 Å, we find the expected intense π → π^*^ transition as the lowest state. However, for the small 6-31G(d) basis, we find that the two lowest states are quite close in energy, thus resulting in shared intensity between the two transitions. Adding diffuse functions, i.e., using 6-31+G(d), or using the larger pcseg-2 basis set, increases the separation between the states and thus largely avoids the issue. Comparing the results obtained using 6-31+G(d), 6-31+G(d,p), and pcseg-2, we observe very small differences for the two lowest states, but the third state differs significantly. This is not necessarily an issue, since we are mainly interested in the lowest intense transition. However, it may be an indication of issues with over-polarization or electron spill-out. Nonetheless, it is clear that the point-charge redistribution distance of 0.5 Å is superior in this case and we therefore only include results based on this choice for the following analyses.

To further investigate the role of the basis set, we take a closer look at the MOs. The six highest occupied MOs and six lowest unoccupied MOs of nCC 14 in vacuum and in the protein (14-DsRed) are provided in Figures S3 and S4, respectively. A comparison of the MOs reveals rather large differences, and in particular the unoccupied MOs depend strongly on whether diffuse functions are used or not, and whether they are determined in vacuum or in the protein. For nCC 14 in vacuum, the diffuse functions, which are present in 6-31+G(d) and 6-31+G(d,p), result in Rydberg-like unoccupied MOs, except for the lowest unoccupied MO (LUMO). Such Rydberg-like orbitals would be expected to be much higher in energy when embedded in an environment (if at all present) due to Pauli repulsion. However, since the PE model does not include Pauli repulsion, the use of diffuse functions or large basis sets is not always straightforward. Indeed, for computations in the protein environment, we observe spurious unoccupied MOs when the 6-31+G(d) and 6-31+G(d,p) were used. Similar effects are not observed, at least not to the same degree, for the unoccupied MOs obtained using pcseg-2 or 6-31G(d), which suggests that the diffuse functions have a negative effect on the MOs when the protein is involved.

Typically, the transition of interest in 2PA processes in FPs is to the lowest-lying excited state, S_1_. The 1PA results for nCC 14 using the functional and basis sets cited above show that this transition is dominated by the highest occupied MO (HOMO) and the LUMO. Therefore, the presence of spurious MOs beyond the HOMO and LUMO might not be considered important and either 6-31+G(d,p) or pcseg-2 can be used. However, the role of the rest of the MOs, especially those with unphysical descriptions, on σ^2PA^, is unknown. They may be important contributors to σ^2PA^, as the expression for the 2PA transition moment involves a sum over all excited states, thus, in principle, involving all MOs. The excitation energies, oscillator strengths, and main MO contributions for S_1_ for all nCCs in vacuum and embedded within the protein matrix are given in Table S3. The excitation to S_1_ in most of the remaining nCCs, besides nCC 14, also involve mainly the HOMO and LUMO. However, in nCC 19, it is HOMO−1 that dominates, while in nCCs 21 and 22 it is primarily HOMO−2. In these cases, the troublesome scenario discussed above, appears to be present even with the redistribution distance of 0.5 Å. The highest occupied and lowest unoccupied MOs of models 19, 21, and 22 are provided in Figures S7–S9. These cases further emphasize that care must be taken when evaluating σ^2PA^ using the PE model and QM/MM approaches in general, particularly, when diffuse functions or large basis sets are used. Besides possible issues present at the QM/MM interface where bonds have been broken, we also suspect that this is a symptom of over-polarization or electron spill-out because of the proximity of the point-charges surrounding the electronic density.

Two-photon absorption cross-sections for the nCCs in vacuum and embedded in the DsRed protein (nCC-DsRed) are shown in Table 1. For the latter, two different approaches were considered: including or excluding local-field effects (denoted PE(+EEF) and PE(−EEF) respectively).

**Table 1:** Two-photon absorption cross-sections (σ^2PA^) for all non-canonical chromophores (nCCs) shown in Figure 1 computed using CAM-B3LYP and 6-31+G(d,p) or pcseg-2 for nCCs in vacuum and nCC-DsRed systems (protein with non-canonical chromophore).

	**Vacuum**			**PE(−EEF)**	**PE(+EEF)**	
**nCC**	**6-31+G(d,p)^†^**	**6-31+G(d,p)^§^**	**pcseg-2^§^**	**6-31+G(d,p)**	**6-31+G(d,p)**	**pcseg-2**
13	19.2	59.7	56.1	23.4	5.7	5.8
14	19.7	58.7	55.4	23.8	5.9	5.8
16a	17.2	32.2	31.1	10.5	2.6	2.7
16b	15.4	48.0	46.4	6.9	1.8	1.9
17	21.7	67.8	64.1	63.7	16.2	16.3
18	29.0	88.1	83.0	55.6	14.1	16.9
19	20.5	76.8	71.8	12.2	3.1	3.1
20	43.9	85.6	82.9	43.3	10.9	15.9
21	15.0	70.6	67.6	4.2	1.1	1.3
22	3.0	6.6	6.8	7.3	2.0	1.5

*For the nCC-DsRed systems, the PE model is used to include the effects from the protein either with effective external field effects [PE(+EEF)] or without [PE(−EEF)]. For comparison, σ^2PA^ results reported by Salem et al. (2016) are included (Salem et al., 2016)*.

† *Salem et al. (2016) results obtained for smaller versions of the chromophores than the ones used here (see Figure 1)*.

§ *Results obtained in this work for the isolated chromophores using the QM/MM-optimized geometries*.

**Figure 7:**
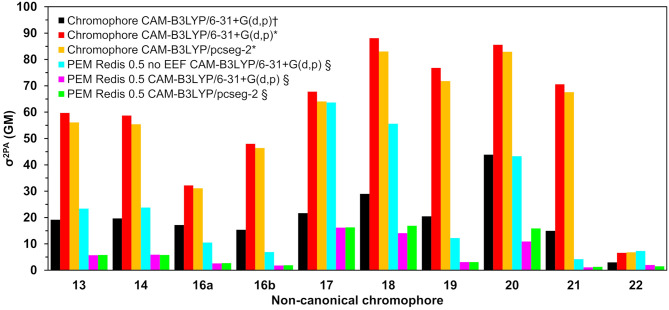
Two-photon absorption cross-sections for all non-canonical chromophores shown in Figure 1 computed (†) in vacuum by Salem et al. (2016) using CAM-B3LYP/6-31+G(d,p) (Salem et al., 2016), in this work using QM/MM-optimized geometries (*) in vacuum, and (§) in protein (using the PE model).

As discussed in the previous subsection, the chromophore geometries, and in particular the tilt and twist angles, obtained in this work differ from the ones determined by Salem et al. (2016) due to the inclusion of the protein effects in the geometry optimization. Moreover, the present models use extended structures. These differences cause the 2PA cross-sections computed in the present work for the chromophores in vacuum to be on average three times larger than the cross-sections previously reported for the same set of chromophores (Salem et al., 2016) (left-hand side of Table 1). However, for the nCC-DsRed models, the effect that the disruption of the planarity, through the increase of tilt and twist angles, has on σ^2PA^ seems to be attenuated by the introduction of the protein. Figure 7 illustrates the variation of σ^2PA^ among the different methods.

The inclusion of local-field effects through the EEF approach [PE(+EEF)] led to an additional reduction in σ^2PA^ on top of the reduction already induced by the direct electrostatic interactions [PE(−EEF)], in comparison with the values obtained in vacuum. For the computations including EEF effects, the σ^2PA^'s are not affected by the basis set choice to any significant extent. In most cases, the difference between results determined using 6-31+G(d,p) and pcseg-2 is 0.1-0.2 GM, and the largest deviation is 5 GM for nCC 20. Previously, List et al. (2016) tested the inclusion of EEF effects on the computation of 1PA and 2PA properties of DsRed. Their study showed that σ^2PA^ of DsRed (30 GM) was 3.5 times smaller than the σ^2PA^ obtained for the same system treated without EEF effects. Here, the σ^2PA^'s obtained for the nCC-DsRed chromophores align with the previous observations, i.e., they are reduced from 3.7 and up to 4.1 times upon inclusion of EEF. In addition, the results obtained here including EEF effects in comparison with the results obtained by Salem et al. (2016) in vacuum and without taking into account the protein in the optimization of the chromophores, are up to 14 times smaller. Unfortunately and somewhat disappointingly, none of the nCC-DsRed models investigated in this work surpass σ^2PA^ of CRQ-DsRed, previously computed by List et al. (2016). The latter is true for all models including nCCs 20 and 21 which showed the largest cross-sections in previous studies (44 and 15 GM, respectively) of the isolated chromophores. In fact, nCC 21 has one of the smallest σ^2PA^, whereas nCC 20 exhibits a σ^2PA^ similar to that of nCCs 17 and 18. The 2PA cross-section for the nCC 20-DsRed system optimized using electrostatic embedding was computed including EEF effects and using the pcseg-2 basis set. The result, 21.9 GM, although larger than what was obtained for nCC 20-DsRed optimized using mechanical embedding, i.e., 15.9 GM, is still inferior to what has been previously predicted in vacuum both in this work and by Salem et al. (2016). It is also smaller than the 2PA cross-section computed for DsRed by List et al. (2016) through a PE scheme. The fact that the difference in 2PA cross-section is rather modest suggests that a refinement of the structures using electrostatic embedding will not have a large impact on the results presented in Table 1, although it stresses the importance of the protein when determining the chromophore structure and multi-photon absorption properties. Similar optimizations of the rest of the systems using electrostatic embedding were not attempted.

## 4. Conclusions

The inclusion of protein effects in the geometry optimization of the nCC-DsRed systems studied here suggest that the identity of the substituent R- in the non-canonical chromophore (see Figure 1) does not have a significant impact on the geometry of the chromophore if such optimizations are carried out in vacuum, i.e., without the protein environment. More realistic pictures of the conformation of the non-canonical chromophores studied in this work needed to be addressed by including the protein environment in QM/MM strategies.

Although the nCC-DsRed systems evaluated in this work involve the same computational models of the non-canonical chromophores previously proposed and investigated by Salem et al. (2016), their 2PA cross-sections have here been evaluated in a more complete way, taking into account protein environment effects, which has not been considered before for these systems. We found that both direct electrostatic interactions and local-field effects have a large impact on the 2PA cross sections. For all chromophores, the 2PA cross sections decreased in comparison with previous studies carried out in vacuum, which highlights the critical role of the environment in the design of new FPs with large 2PA cross-sections. The results obtained in this work suggest that the choice of basis set when using QM/MM models should be done carefully, as diffuse functions can result in spurious molecular orbitals, whose impact on the σ^2PA^ computation has not been evaluated extensively.

In this work, the DsRed protein was chosen as the protein host for the set of non-canonical chromophores. Future work could involve the evaluation of 2PA properties of selected nCCs in other RFP hosts and/or a tailored environment, where amino acids surrounding the chromophore can be modified or substituted to tune its 1PA and 2PA properties (List et al., 2012b; Beerepoot et al., 2014b; Nifosì et al., 2019). Water molecules in the immediate surroundings of the chromophore can play a role in the absorption properties of fluorescent proteins (Zhang et al., 2011; Konold et al., 2014, 2016; Faraji and Krylov, 2015; Şimşek and Brown, 2018). However, we do not expect that the 2PA cross-sections obtained here would change drastically such that they surpass the 2PA cross-section limit exhibited by existing FPs. That said, it would be useful to establish their role, if present, on 2PA as well as examine the effects from solvent and conformational sampling.

## Data Availability Statement

The datasets generated in this study can be found in the repository Dataset for the article: Two-photon Absorption Cross-sections in Fluorescent Proteins Containing Non-canonical Chromophores Using Polarizable QM/MM (https://doi.org/10.6084/m9.figshare.11886981.v1).

## Author Contributions

The study was conceived by MR-T and AB. MR-T carried out all simulations under the primary guidance of JO with input from AB. The manuscript was written by MR-T and JO with editorial and scientific contributions from AB.

## Conflict of Interest

The authors declare that the research was conducted in the absence of any commercial or financial relationships that could be construed as a potential conflict of interest.

## Supplementary Material

Supplementary Material includes tilt and twist angles computed for the nCC models optimized using the ONIOM scheme, molecular orbitals for nCCs 14, 19, 21, and 22 in vacuum and using the PEM scheme. Excitation energies and oscillator strengths are also provided.
