# Supplementary material for: Two-Photon Absorption Cross-Sections in Fluorescent Proteins Containing Non-canonical Chromophores Using Polarizable QM/MM
Source: Front Mol Biosci. 2020 Jun 12;7:111. doi: 10.3389/fmolb.2020.00111 (PMC7303285; doi:10.3389/fmolb.2020.00111)
Supplement: Supplementary file 1 [file Data_Sheet_1.PDF]

Supplementary Material

FIGURES

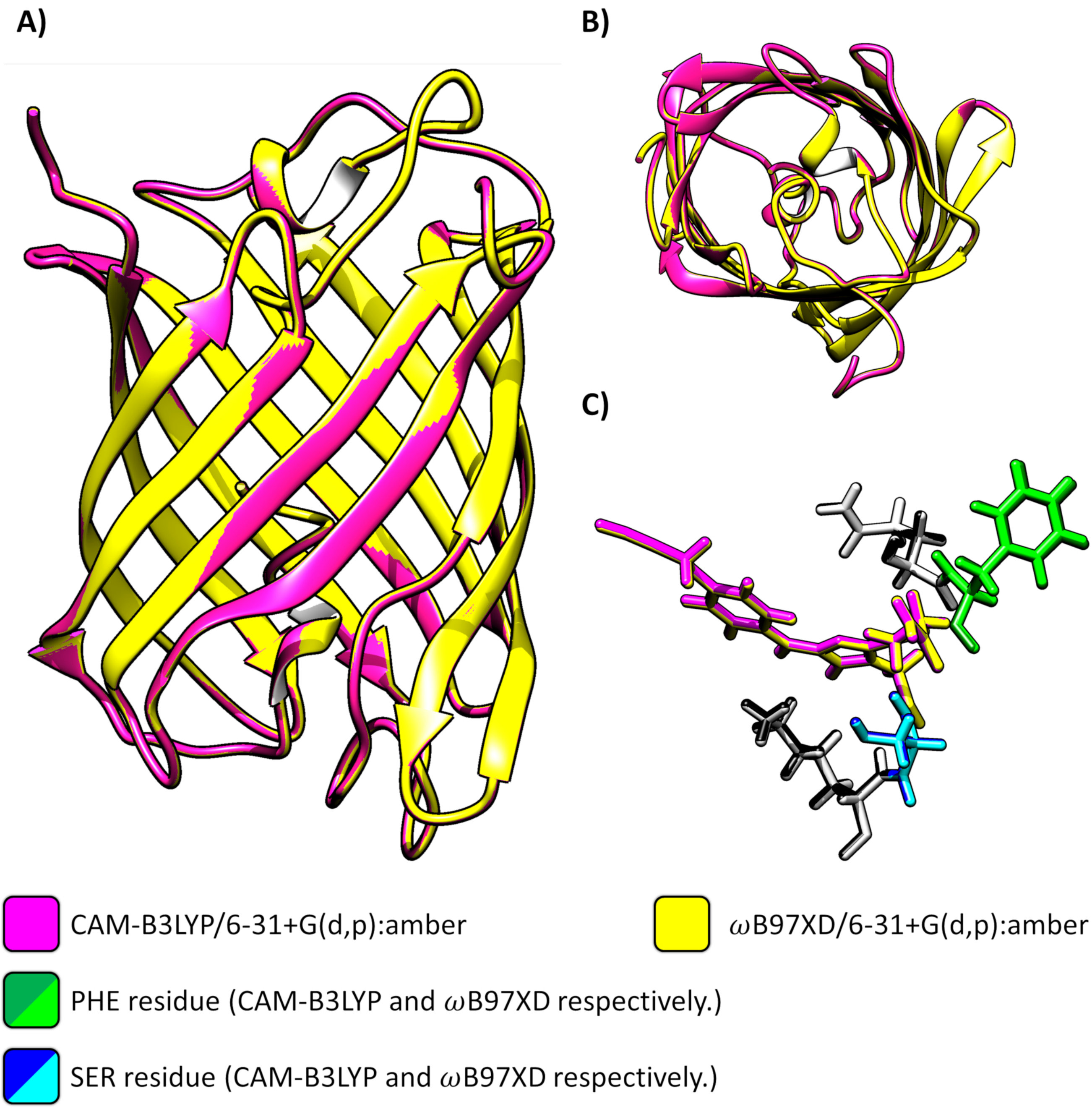

Figure S1. For system nCC-14, A) side-view and B) top-view of overlapped structures of 1ZGO protein, and C) overlapped chromophore structures optimized using CAM-B3LYP/6-31+G(d,p) and ωB97XD methods within ONIOM scheme.

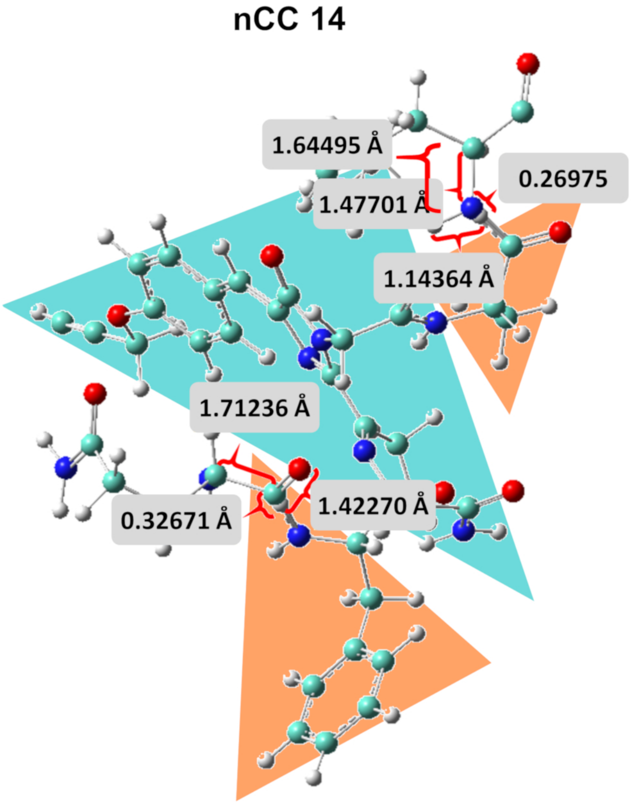

Figure S2. Distances between dangling hydrogen atoms at the N-terminal and C-terminal side of the SER-nCC-PHE QM area and selected atoms in the MM zone.

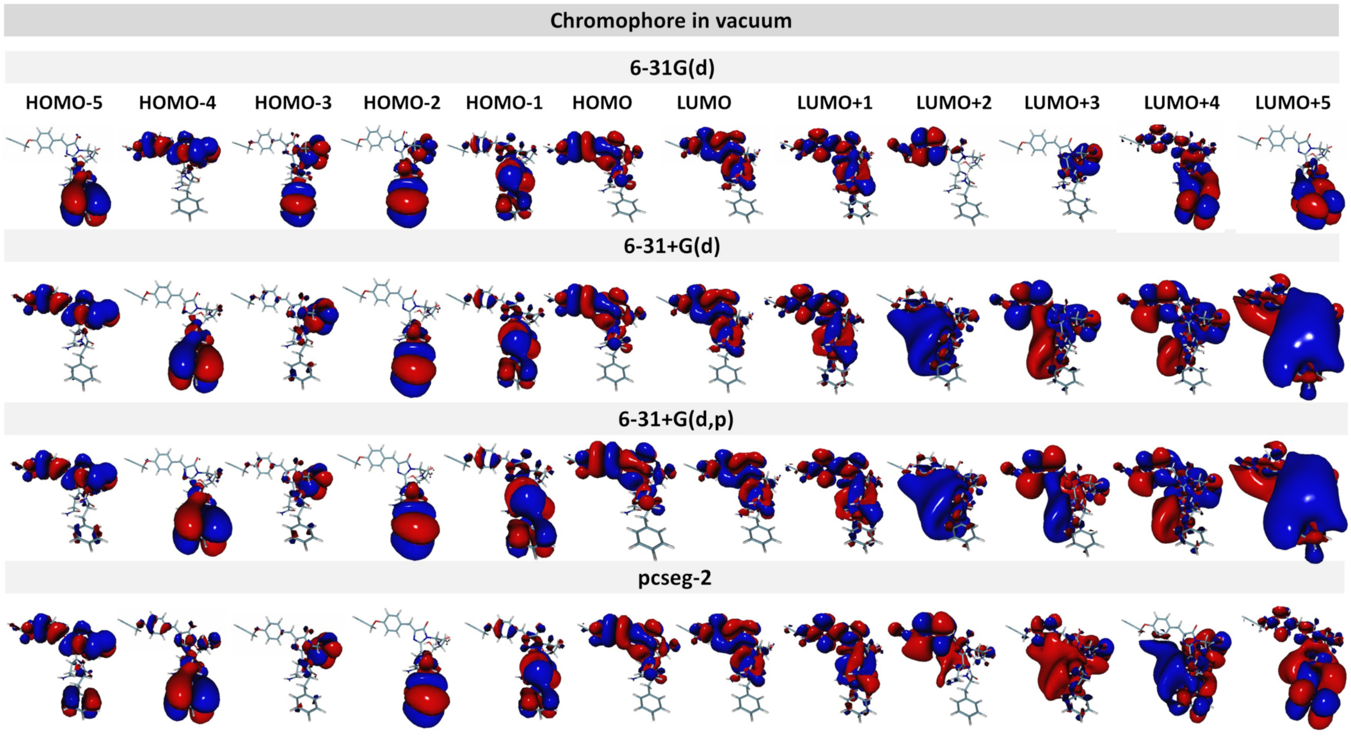

Figure S3. Molecular orbitals computed for the isolated nCC 14 using CAM-B3LYP functional and different basis sets. Plots were obtained using Molden software [Schaftenaar and Noordik (2000); Schaftenaar et al. (2017)]. Isovalue = 0.01.

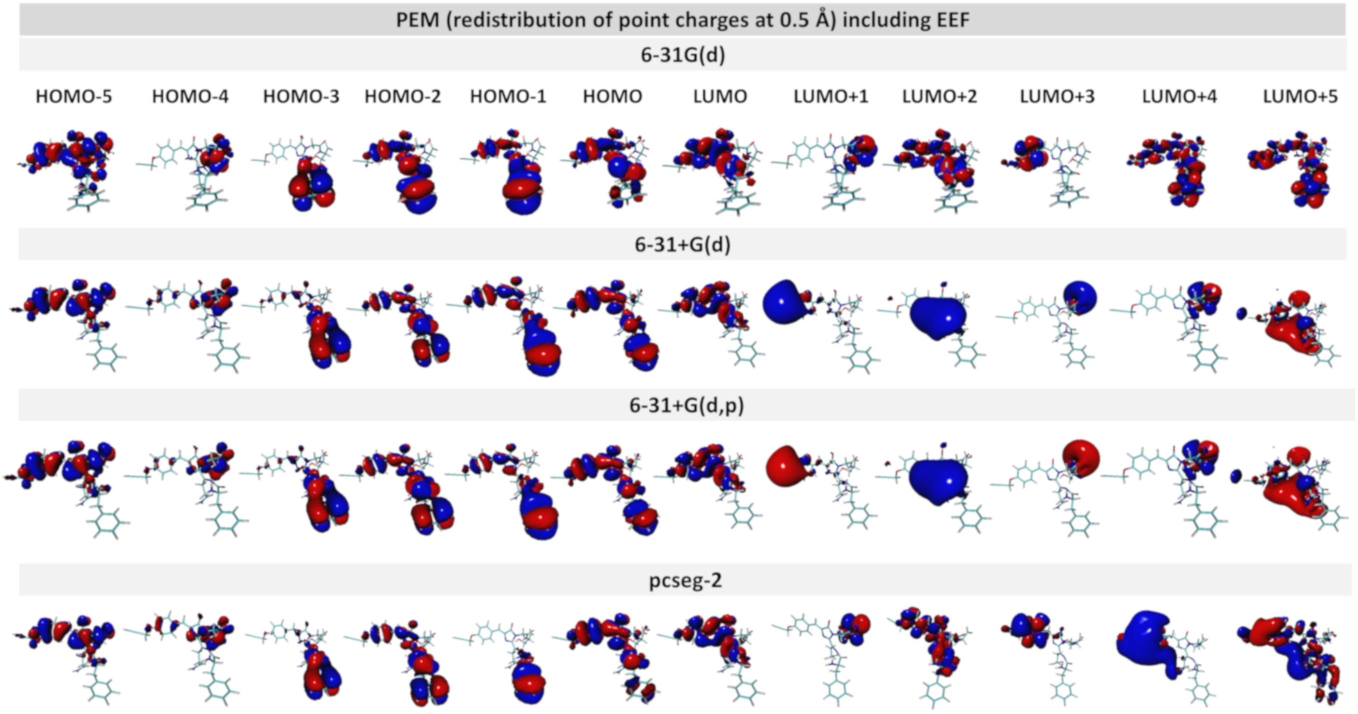

Figure S4. Molecular orbitals computed for the 14-DsRed system using CAM-B3LYP functional and different basis sets. Charge redistribution at 0.5 Å was used. Plots were obtained using Molden software [Schaftenaar and Noordik (2000); Schaftenaar et al. (2017)]. Isovalue = 0.005.

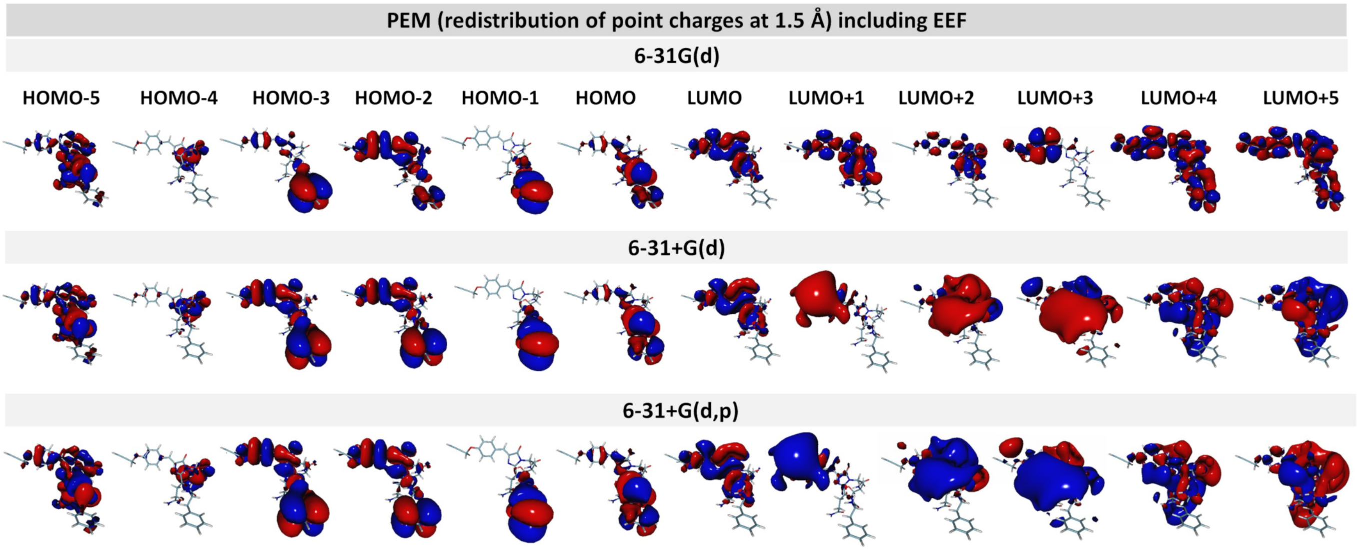

Figure S5. Molecular orbitals computed for the 14-DsRed system using CAM-B3LYP functional and different basis sets. Charge redistribution at 1.5 Å was used. Plots were obtained using Molden software [Schaftenaar and Noordik (2000); Schaftenaar et al. (2017)]. Isovalue = 0.005.

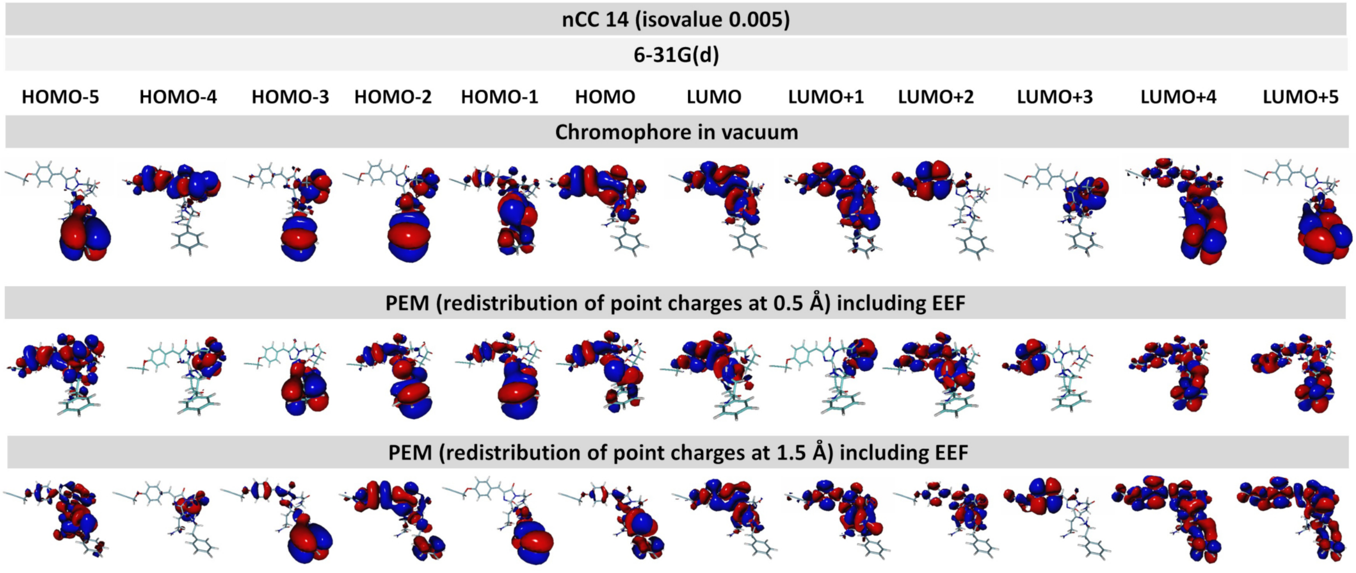

Figure S6. Molecular orbitals computed using CAM-B3YP/6-31G(d) method for the nCC 14, i) in vacuum and ii) in protein using charge redistribution at 0.5 Å and ii) in protein using charge redistribution at 1.5 Å. Plots were obtained using Molden software [Schaftenaar and Noordik (2000); Schaftenaar et al. (2017)]. Isovalue = 0.005.

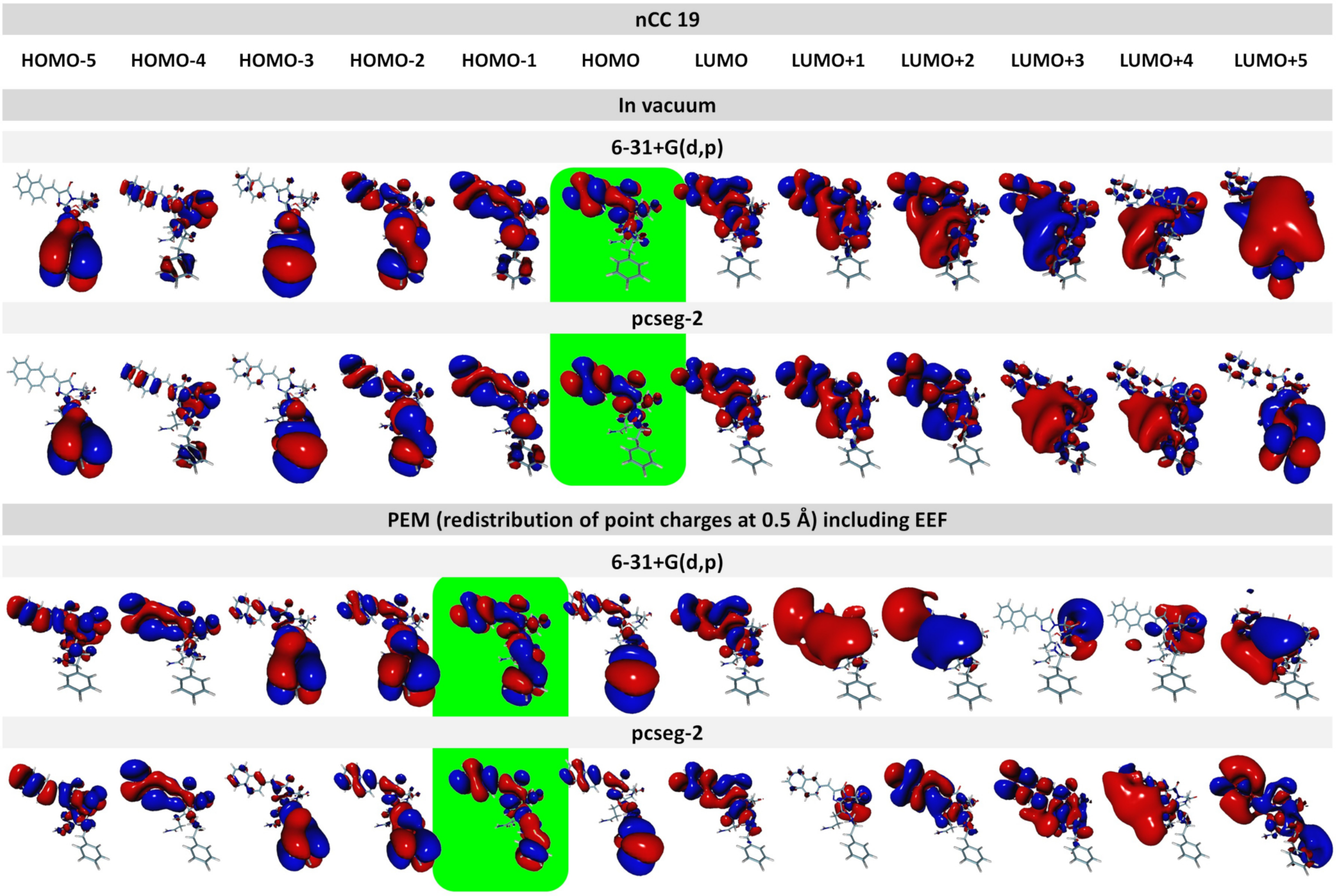

Figure S7. Molecular orbitals computed for the nCC 19 and the 19-DsRed system using CAM-B3LYP and different basis sets. The shift between states of orbitals obtained in protein (through the PE model) and in vacuum is stressed in green. Plots were obtained using Molden software [Schaftenaar and Noordik (2000); Schaftenaar et al. (2017)]. Isovalue = 0.005

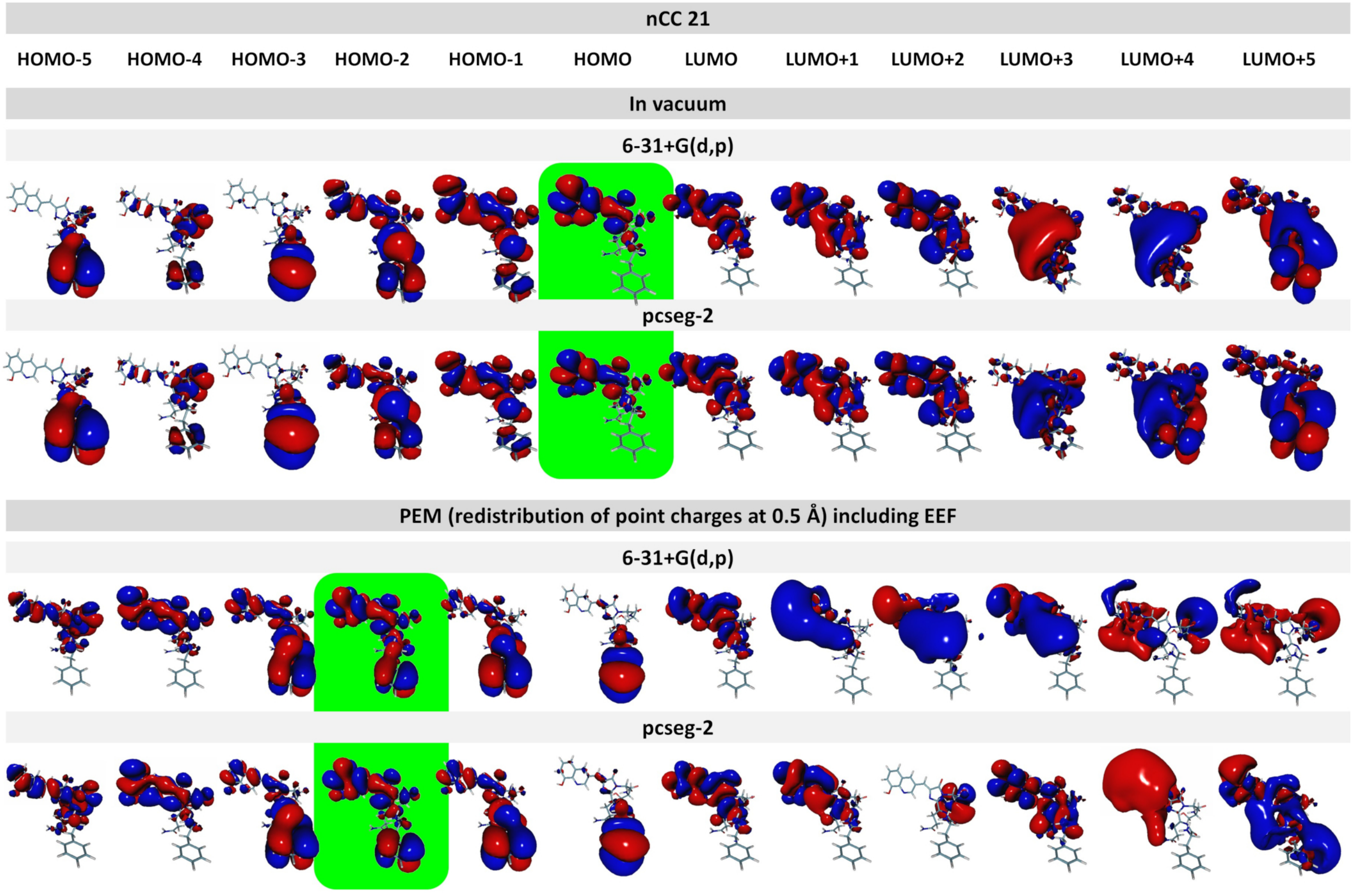

Figure S8. Molecular orbitals computed for the nCC 21 and the 21-DsRed system using CAM-B3LYP and different basis sets. The shift between states of orbitals obtained in protein (through the PE model) and in vacuum is stressed in green. Plots were obtained using Molden software [Schaftenaar and Noordik (2000); Schaftenaar et al. (2017)]. Isovalue = 0.005.

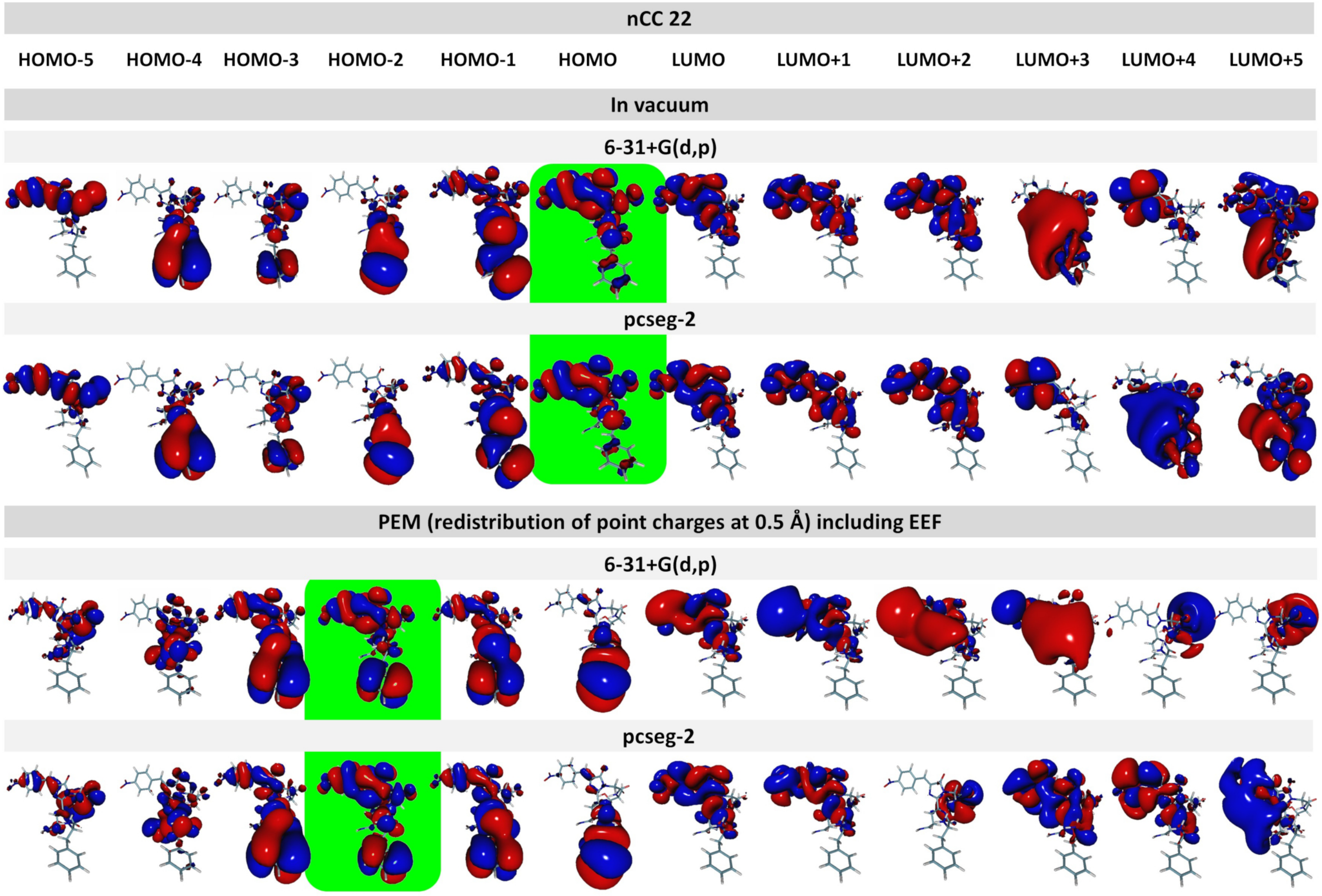

Figure S9. Molecular orbitals computed for the nCC 22 and the 22-DsRed system using CAM-B3LYP and different basis sets. The shift between states of orbitals obtained in protein (through the PE model) and in vacuum is stressed in green. Plots were obtained using Molden software [Schaftenaar and Noordik (2000); Schaftenaar et al. (2017)]. Isovalue = 0.005.

TABLES

Table S1. Tilt and twist angles, and angle differences between structures optimized using ONIOM scheme CAM-B3LYP/6-31+G(d,p):Amber, PBE0/6-31+G(d,p) in vacuum [Salem et al. (2016)], and the crystal structure [Tubbs et al. (2005)].

	PBE0		CAM-B3LYP		CAM-B3LYP–PBE0		CAM-B3LYP–crystal		PBE0–crystal	
nCC	Tilt (°)	Twist (°)	Tilt (°)	Twist (°)	Tilt (°)	Twist (°)	Tilt (°)	Twist (°)	Tilt (°)	Twist (°)
13	0.17	-0.01	-1.90	-0.67	-2.07	-0.66	-6.70	-3.98	-4.63	-3.32
14	-0.57	-1.12	0.84	-4.59	1.41	-3.46	-3.96	-7.89	-5.37	-4.43
16a	-0.36	-1.33	-1.55	-3.92	-1.19	-2.58	-6.35	-7.23	-5.16	-4.64
16b	0.21	-0.08	-3.46	-5.04	-3.67	-4.96	-8.26	-8.35	-4.59	-3.39
17	-0.53	-1.36	-5.89	-16.80	-5.36	-15.45	-10.69	-20.11	-5.34	-4.67
18	-0.40	-1.39	-3.93	-0.13	-3.53	1.26	-8.74	-3.44	-5.20	-4.70
19	-0.75	-3.71	-1.39	2.11	-0.64	5.82	-6.20	-1.20	-5.56	-7.02
20	-0.09	6.12	0.27	8.29	0.36	2.17	-4.53	4.98	-4.90	2.82
21	-0.15	-2.61	-2.94	-2.30	-2.78	0.30	-7.74	-5.61	-4.96	-5.92
22	0.23	0.11	-1.63	-1.76	-1.87	-1.86	-6.44	-5.06	-4.57	-3.20
20*			-1.59	11.8	-1.49	5.05	-6.39	7.87		

	Crystal structure	
	Tilt (°)	Twist (°)
CRQ	4.81	3.31

*Using an electrostatic embedding scheme.

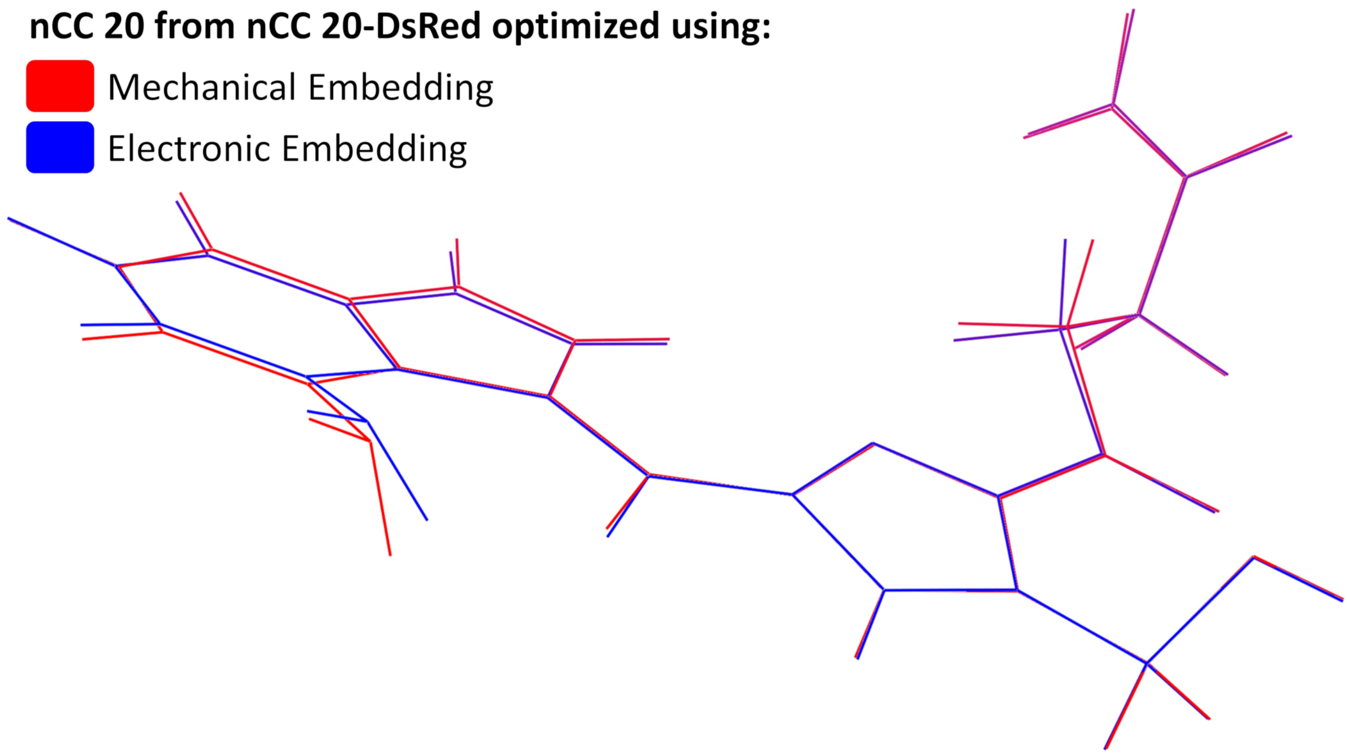

Figure S10. Depiction of the structural differences in nCC 20 between optimizing nCC 20-DsRed using mechanical embedding and electrostatic embedding.

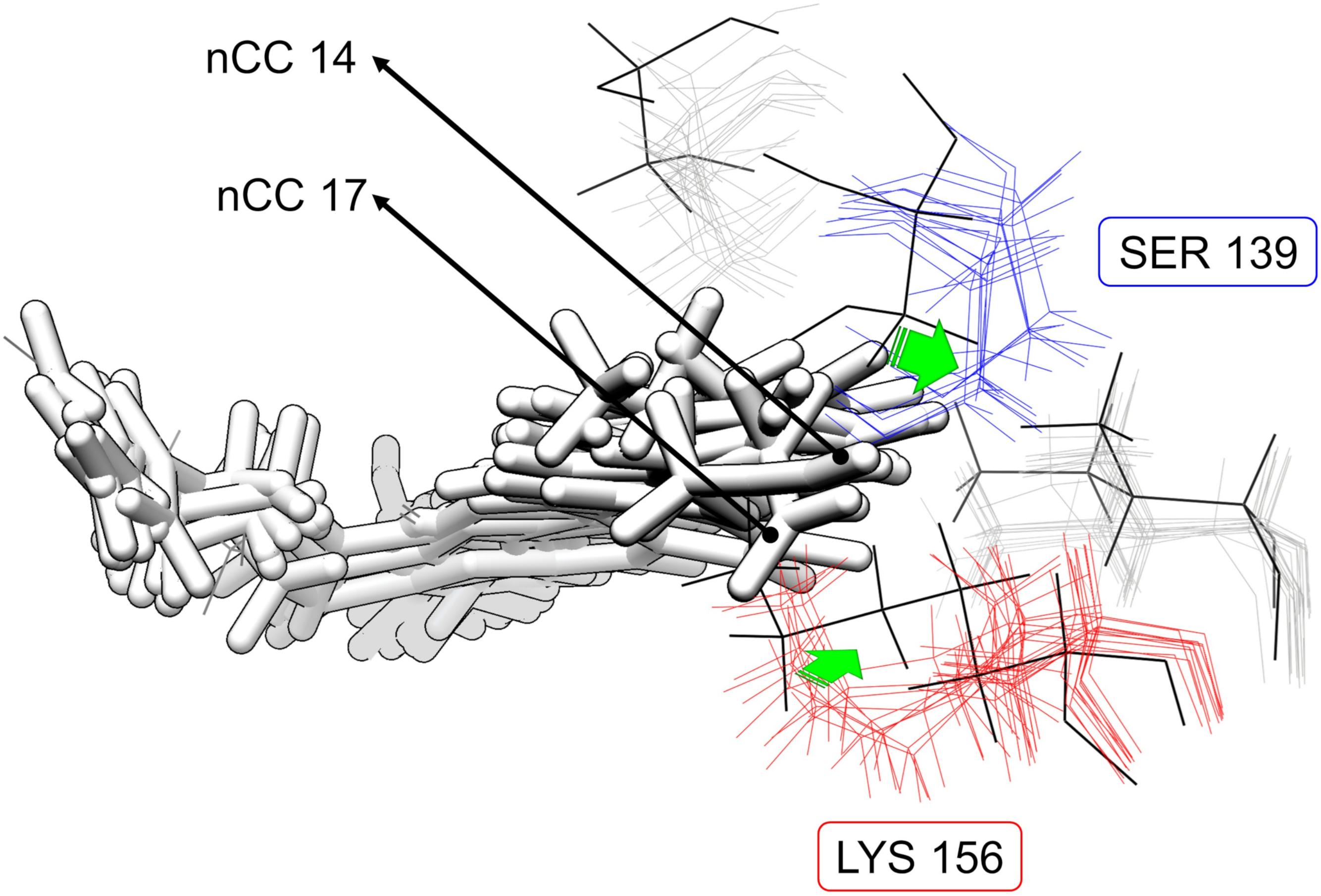

Figure S11. Structural changes of selected residues, SER 139 (or 146 in the 1ZGO indexing) and LYS 156 (or 163 in the 1ZGO indexing), non-covalently bonded to the non-canonical chromophore. The green arrows indicate the contraction of serine and lysine towards the chromophore as the identity of the chromophore is changed. 1ZGO crystal structure is represented by the wire structure in black.

Table S2. For system 14-DsRed, excitation energies, oscillator strengths, two-photon absorption cross-sections, and molecular orbitals of the first three excited states computed using different basis sets and redistributing point charges that are within 0.5 and 1.5 Å of a QM atom (end removing all other parameters).

**nCC 14**				
**Redistributing point charges within 0.5 Å**				
	ω (eV)	OS	MOs	σ^{2PA} (GM)
6-31G(d)				
S_1	3.433	0.700	HOMO–LUMO	4.0E+00
S_2	3.535	0.103	HOMO-2–LUMO	4.1E+00
S_3	3.969	0.002	HOMO-9–LUMO	7.0E-01
6-31+G(d)				
S_1	3.339	0.762	HOMO–LUMO	5.9E+00
S_2	3.568	0.012	HOMO-2–LUMO	2.8E+00
S_3	3.777	0.013	HOMO–LUMO+1	4.0E+00
6-31+G(d,p)				
S_1	3.336	0.760	HOMO–LUMO	5.9E+00
S_2	3.569	0.011	HOMO-2–LUMO	2.7E+00
S_3	3.774	0.014	HOMO–LUMO+1	4.0E+00
pcseg-2				
S_1	3.338	0.778	HOMO–LUMO	5.8E+00
S_2	3.565	0.013	HOMO-2–LUMO	2.5E+00
S_3	3.966	0.002	HOMO-6–LUMO	1.1E+00
**Redistributing point charges within 1.5 Å**				
	ω (eV)	OS	MOs	σ^{2PA} (GM)
6-31G(d)				
S_1	3.245	0.051	HOMO–LUMO	1.8E+00
S_2	3.474	0.789	HOMO-2–LUMO	9.0E+00
S_3	3.987	0.001	HOMO-10–LUMO	7.6E-01
6-31+G(d)				
S_1	3.260	0.186	HOMO–LUMO	1.6E+00
S_2	3.374	0.615	HOMO-3–LUMO	1.0E+01
S_3	3.861	0.013	HOMO-3–LUMO+1	4.4E+00
6-31+G(d,p)				
S_1	3.262	0.203	HOMO–LUMO	1.6E+00
S_2	3.372	0.596	HOMO-2–LUMO	1.0E+01
S_3	3.857	0.013	HOMO-2–LUMO+1	4.4E+00

Table S3. For transition S_1 of all non-canonical chromophores (nCC) used in this work, excitation energies (ω), molecular orbitals involved in the transition, and oscillator strengths computed for both, chromophore in vacuum and protein-nCC systems using QM/MM polarized embedding (PE) model.

			**PE**			**Chromophore (vacuum)**	
**nCC**	**Basis set**	ω **(eV)**	**Transition**	**OS**	ω **(eV)**	**Transition**	**OS**
13	6-31+G(d,p)	3.273	HOMO - LUMO	0.654	3.242	HOMO - LUMO	0.612
13	pcseg-2	3.276	HOMO - LUMO	0.655	3.228	HOMO - LUMO	0.609
14	6-31G(d)	3.433	HOMO - LUMO	0.700	3.384	HOMO - LUMO	0.693
14	6-31+G(d)	3.339	HOMO - LUMO	0.762	3.320	HOMO - LUMO	0.724
14	6-31+G(d,p)	3.336	HOMO - LUMO	0.760	3.317	HOMO - LUMO	0.723
14	pcseg-2	3.338	HOMO - LUMO	0.778	3.302	HOMO - LUMO	0.724
16a	6-31+G(d,p)	3.369	HOMO - LUMO	0.581	3.384	HOMO - LUMO	0.539
16a	pcseg-2	3.367	HOMO - LUMO	0.599	3.363	HOMO - LUMO	0.543
16b	6-31+G(d,p)	3.401	HOMO - LUMO	0.658	3.375	HOMO - LUMO	0.662
16b	pcseg-2	3.396	HOMO - LUMO	0.676	3.353	HOMO - LUMO	0.665
17	6-31+G(d,p)	3.245	HOMO - LUMO	0.894	3.242	HOMO - LUMO	0.788
17	pcseg-2	3.244	HOMO - LUMO	0.920	3.223	HOMO - LUMO	0.783
18	6-31+G(d,p)	3.172	HOMO - LUMO	0.778	3.179	HOMO - LUMO	0.737
18	pcseg-2	3.198	HOMO - LUMO	0.830	3.167	HOMO - LUMO	0.733
19	6-31+G(d,p)	3.296	HOMO$-$1 - LUMO	0.890	3.285	HOMO - LUMO	0.856
19	pcseg-2	3.290	HOMO$-$1 - LUMO	0.901	3.268	HOMO - LUMO	0.853
20	6-31+G(d,p)	2.975	HOMO - LUMO	0.638	3.023	HOMO - LUMO	0.625
20	pcseg-2	3.043	HOMO - LUMO	0.759	3.010	HOMO - LUMO	0.625
21	6-31+G(d,p)	3.328	HOMO$-$2 - LUMO	0.854	3.346	HOMO - LUMO	0.816
21	pcseg-2	3.323	HOMO$-$2 - LUMO	0.869	3.325	HOMO - LUMO	0.816
22	6-31+G(d,p)	3.294	HOMO$-$2 - LUMO	0.535	3.366	HOMO - LUMO	0.487
22	pcseg-2	3.292	HOMO$-$2 - LUMO	0.549	3.347	HOMO - LUMO	0.490
			Electrostatic Embedding:				
20	pcseg-2	2.99	HOMO - LUMO	0.73	2.90	HOMO - LUMO	0.56

REFERENCES

Schaftenaar G, Noordik JH. Molden: a pre- and post-processing program for molecular and electronic structures*. *J. Comput. Aided Mol. Des.* **14** (2000) 123–134. doi:10.1023/A:1008193805436.

Schaftenaar G, Vlieg E, Vriend G. Molden 2.0: quantum chemistry meets proteins. *Journal of Computer-Aided Molecular Design* **31** (2017) 789–800. doi:10.1007/s10822-017-0042-5.

Salem MA, Twelves I, Brown A. Prediction of two-photon absorption enhancement in red fluorescent protein chromophores made from non-canonical amino acids. *Phys. Chem. Chem. Phys.* **18** (2016) 24408–24416. doi:10.1039/C6CP03865D.

Tubbs JL, Tainer JA, Getzoff ED. Crystallographic structures of discosoma red fluorescent protein with immature and mature chromophores: Linking peptide bond trans–cis isomerization and acylimine formation in chromophore maturation. *Biochemistry* **44** (2005) 9833–9840. doi:10.1021/bi0472907. PMID: 16026155.
